# Where do bright ideas occur in our brain? Meta-analytic evidence from neuroimaging studies of domain-specific creativity

**DOI:** 10.3389/fpsyg.2015.01195

**Published:** 2015-08-11

**Authors:** Maddalena Boccia, Laura Piccardi, Liana Palermo, Raffaella Nori, Massimiliano Palmiero

**Affiliations:** ^1^Department of Psychology, “Sapienza” University of RomeRome, Italy; ^2^Neuropsychology Unit, I.R.C.C.S. Fondazione Santa Lucia of RomeRome, Italy; ^3^Department of Life, Health and Environmental Sciences, University of L’AquilaL’Aquila, Italy; ^4^School of Life and Health Sciences, Aston UniversityBirmingham, UK; ^5^Department of Psychology, University of BolognaBologna, Italy

**Keywords:** creativity, musical improvisation, divergent thinking, verbal processing, visuo-spatial processing, idea generation, open-ended problems, executive functions

## Abstract

Many studies have assessed the neural underpinnings of creativity, failing to find a clear anatomical localization. We aimed to provide evidence for a multi-componential neural system for creativity. We applied a general activation likelihood estimation (ALE) meta-analysis to 45 fMRI studies. Three individual ALE analyses were performed to assess creativity in different cognitive domains (Musical, Verbal, and Visuo-spatial). The general ALE revealed that creativity relies on clusters of activations in the bilateral occipital, parietal, frontal, and temporal lobes. The individual ALE revealed different maximal activation in different domains. Musical creativity yields activations in the bilateral medial frontal gyrus, in the left cingulate gyrus, middle frontal gyrus, and inferior parietal lobule and in the right postcentral and fusiform gyri. Verbal creativity yields activations mainly located in the left hemisphere, in the prefrontal cortex, middle and superior temporal gyri, inferior parietal lobule, postcentral and supramarginal gyri, middle occipital gyrus, and insula. The right inferior frontal gyrus and the lingual gyrus were also activated. Visuo-spatial creativity activates the right middle and inferior frontal gyri, the bilateral thalamus and the left precentral gyrus. This evidence suggests that creativity relies on multi-componential neural networks and that different creativity domains depend on different brain regions.

## Introduction

The ability to form novel ideas is crucial for human civilization, progress, and innovation. Creativity has been defined as “the introduction of something innovatively new and positive for society that goes beyond the familiar and accepted” (Zaidel, 2014, p. 1) and concerns many domains of human activities (Gonen-Yaacovi et al., 2013), such as science, technology, economy, and arts. However, creativity concerns not only exceptional realizations, such as scientific discoveries or the production of artworks, but also everyday activities, such as finding new solutions and thinking away from ordinary ideas. Furthermore, creativity includes the appropriateness (Sternberg and Lubart, 1999; Runco and Jaeger, 2012) of the new ideas and solutions. The product of creativity must, in fact, involve an actual use in a specific context, rather than a hypothetical use.

Evolution has strongly fostered creativity. Bio-social pressures toward creativity are thought to have shaped the evolution of the human brain (Zaidel, 2014). Previous neuroimaging studies failed to find a clear neuroanatomical localization of creative processes (for a review, see Dietrich and Kanso, 2010; Mihov et al., 2010): creativity does not appear to critically rely on any single brain area and it is not especially associated with the right or left brain hemispheres (Dietrich and Kanso, 2010). The failure to find any clear neuroanatomical localization is likely due to the fact that creativity is a multifaceted process, which is supported by high-level mental operations, both independent (for example, abstraction; Welling, 2007) and dependent (for example, domain-specific operations) on the specific domains of knowledge. Palmiero et al. (2010) found that verbal creativity is mostly domain-specific, but can also be affected by processes in the visual domain, whereas visual creativity is domain- and task-specific. Various different approaches and tasks have been used to explore creativity. Some rely on the ability to find one correct solution to closed problems, such as insight problem solving, others rely on the ability to find new, appropriate, and different answers to open-ended problems, such as divergent thinking, creative cognition, and artistic creativity.

The divergent thinking approach was introduced by Guilford (1950, 1967). The Alternative Uses Task (AUT), which requires individuals to generate as many different alternative uses of a specific object (e.g., a brick) as possible, was initially used to assess divergent thinking in terms of ideational fluency (the number of ideas), flexibility (the number of categories that encompass ideas), originality (infrequency of ideas), and elaboration (the number of details added to basic ideas). In the wake of Guilford’s (1950, 1967) work, Torrance (1974) developed the Torrance Test of Creative Thinking (TTCT), which was aimed at measuring divergent thinking in verbal and visual forms. Recently, the idea that divergent thinking is an indicator of creative potential without guaranteeing actual creative achievement has emerged (Runco and Acar, 2012). In addition, divergent thinking is supported by convergent thinking for the evaluation of the novelty of ideas (Cropley, 2006).

The creative cognition approach is mainly based on the ‘Geneplore’ model (Finke et al., 1992; Smith et al., 1995) that focuses on mental operations involved in visual creativity. This approach assumes that generative (e.g., memory retrieval, mental synthesis) and exploratory (e.g., conceptual interpretation, functional inference) processes support creativity. Specifically, generative processes support the construction of visual pre-inventive ideas, whereas exploratory processes examine and interpret the pre-inventive ideas. The Geneplore model was operationalized by means of the creative synthesis task (Finke, 1990, 1996), which allows individuals to imagine and manipulate visual elements (e.g., square, wire, and bracket), in order to create an object belonging to a specific category. Independent judges are then asked to score the inventions on the basis of different criteria, such as originality and practicality, according to the Consensual Assessment Technique developed by Amabile (1983). Investigations based on this Model have highlighted, among other things, that mental imagery – a complex cognitive process arising when perceptual information is accessed from the memory, giving rise to the experience of “seeing with the mind’s eye” (Kosslyn, 1980; Farah, 1989) – seems to have a pivotal role in directing creative processes. This is confirmed by several scientific studies (e.g., Finke, 1990; Palmiero et al., 2011).

Artistic creativity has been described in terms of an altered state of mind, beyond conscious awareness (Dietrich, 2004). This makes it more difficult to investigate artistic creativity and its cognitive and neural underpinnings. In these last years, the neural processes underlying free generation and selection of possible alternatives have been investigated by using simpler model behaviors, which resemble valid examples of creativity in musical (Bengtsson et al., 2007) and visual domains (Kowatari et al., 2009).

Despite the variety of creativity domains, and of the approaches and tasks used, many pivotal processes supporting creativity can be identified. First, executive functions, such as planning, working memory, attention, and semantic memory retrieval are required. These processes facilitate both the selection (Gabora, 2010) and evaluation of the utility of novel ideas (Howard-Jones and Murray, 2003). Accordingly, the prefrontal cortex recruitment (e.g., the dorsolateral prefrontal cortex – DLPFC) has been widely shown as being involved in verbal divergent thinking based on ideational fluency (e.g., Carlsson et al., 2000; Seger et al., 2000), story generation (Bechtereva et al., 2004; Howard-Jones et al., 2005), metaphor production (Benedek et al., 2014a), creative objects production (Ellamil et al., 2012; Aziz-Zadeh et al., 2013), visual art (Kowatari et al., 2009; Huang et al., 2013), and musical improvisation (e.g., de Manzano and Ullén, 2012; Villarreal et al., 2013; Pinho et al., 2014). Second, creativity also relies on an associative mode of processing (Ellamil et al., 2012), which is supported by the default mode network (e.g., the medial prefrontal and posterior cingulate cortices, temporoparietal junction, part of the medial temporal lobe and the inferior parietal cortex – Buckner et al., 2008). Interestingly, the default mode network is activated during different creativity performances (e.g., Bechtereva et al., 2004; Howard-Jones et al., 2005). Third, memory processes also support creativity. The medial temporal lobe (hippocampal and parahippocampal regions) is recruited during verbal divergent thinking (Fink et al., 2009), creative writing (Shah et al., 2013), metaphor production (Benedek et al., 2014a), visual creativity (Ellamil et al., 2012), and visual art (Kowatari et al., 2009). According to Dietrich (2004) the connections between the dorsolateral prefrontal cortex and the temporal, occipital and parietal cortices, sites of long-term memory storage (e.g., Gilbert, 2001), are essential for creativity. Furthermore, brain areas generally involved in mental imagery, such as the middle occipital gyrus and parietal lobes (Sack et al., 2005; Olivetti Belardinelli et al., 2009; Boccia et al., 2015), can be recruited during creativity, suggesting a top–down control on the construction of the images, even if visual information is not directly manipulated.

Here we aimed to find the neural correlates of creativity in general and those more strictly correlated with the cognitive domain. In the present study creativity is operationally defined as the ability to find new, appropriate, and different answers to open-ended problems, focusing on the idea that a valid assessment of creativity requires tasks that are sufficiently open-ended to encourage divergent production (Green et al., 2015). We applied a general activation likelihood estimation (ALE) meta-analysis of fMRI experiments on creativity based on open-ended mental problems, to find converging evidence for a neural network for creativity in the human brain. Furthermore, three individual ALE analyses were performed to assess whether creativity in different domains (i.e., Musical, Verbal, and Visuo-spatial) involves different brain areas. The decision to explore Musical, Verbal, and Visuo-spatial creativity was made because these were the only domains in which the number of experiments and critical contrasts was sufficient for statistical testing.

Following Dietrich and Kanso’s (2010, p. 822) idea of functionally subdividing different types of creativity “to make creativity tractable in the brain,” we hypothesized that, beyond a common pattern of brain activations generally underpining idea generation in the attempt to solve open-ended problems, different brain regions underpin different domains of creativity and that a multi-componential neural system underpins creative thinking in humans.

## Materials and Methods

### Inclusion Criteria for Papers

A systematic method was adopted to review the literature. The search was carried out with the aid of PubMed, using the following string: “creativity and fMRI.” A total of 56 studies were found.

Our *a priori* inclusion criteria for papers were: (1) Inclusion of whole-brain analysis performed using functional magnetic resonance imaging (fMRI); thus, we excluded positron emission tomography (PET) studies, electrophysiology studies and papers that reported only results from ROI analysis. (2) Provision of coordinates of activation foci, both in the Montreal Neurological Institute (MNI) and the Talairach reference space. (3) All participants in the studies had to be young and healthy. (4) Only studies focusing on open-ended mental problems were included in the meta-analysis; thus we excluded studies exploring neural correlates of idea generation based on closed-ended problems, such as problems based on the combination of remote semantic associations, which generally underpin insight (a stage of the creative process) rather than creativity *per se*. This decision was made following the idea that the “rigorous investigation of creativity requires tasks that are suitable for quantified psychometrics but also sufficiently open-ended to be construct-valid assays of creativity (i.e., they must allow freedom for divergent production)” (Green et al., 2015, p. 924). (5) Only group studies involving a sample size of at least five participants were included. (6) There could be no pharmacological manipulation. (7) Only activation foci were considered. Thus, studies reporting only deactivation foci were excluded from our meta-analysis. (8) Only peer-reviewed original articles were included. Using these criteria we selected 24 articles. The studies are summarized in **Table 1**, where the subdivision according to domains (Musical, Verbal, and Visuo-spatial) is also shown (see below).

**Table 1:** List of papers included in the meta-analysis for each domain.

Paper	Experiments	Subjects	Approach	Task
**Musical domain**				
Bengtsson et al. (2007)	1	11	Artistic creativity	Melody Improvisation (Piano)
Berkowitz and Ansari (2008)	3	12	Artistic creativity	Melody and Rhythmic Improvisation
de Manzano and Ullén (2012)	2	18	Artistic creativity	Melody Improvisation
Limb and Braun (2008)	2	6	Artistic creativity	Melody Improvisation (Scale/Jazz)
Liu et al. (2012)	1	12	Artistic creativity	Lyric Improvisation
Pinho et al. (2014)	2	39	Artistic creativity	Musical Improvisation (Classical/Jazz Piano): Tonal/Atonal; Happy/Fearful
Villarreal et al. (2013)	2	24	Artistic creativity	Rhythmic Creation A synthetic sound was used with a timbre similar to sound produced by the cymbal
**Verbal domain**				
Abraham et al. (2012)	1	19	Divergent Thinking	Alternative Uses
Abraham et al. (2014)	4	28	Divergent Thinking	Alternative Uses
Benedek et al. (2014a)	2	35	Divergent Thinking	Metaphor production
Benedek et al. (2014b)	3	28	Divergent Thinking	Alternative Uses
^∗^Chrysikou and Thompson-Schill (2011)	1	24	Divergent Thinking	Alternative Uses
Fink et al. (2010)	1	31	Divergent Thinking	Alternative Uses in three conditions: Standard, Incubation, Exposure to other people’s ideas
Fink et al. (2012)	3	24	Divergent Thinking	Alternative Uses stimulated by other people’s ideas
^∗^Green et al. (2015)	1	55	Divergent Thinking	Verb Generation
^∗^Howard-Jones et al. (2005)	2	8	Divergent Thinking	Creative Story Generation
Seger et al. (2000)	1	7	Divergent Thinking	Unusual Verb Generation cued by novel and repeated nouns
^∗^Shah et al. (2013)	3	28	Divergent Thinking	Planning and Writing a Story
^∗^Zhang et al. (2014)	2	18	Divergent Thinking	Inventive Conception Generation involving remote semantic relatedness
**Visuo-spatial domain**				
^∗^Asari et al. (2008)	1	68	Divergent Thinking	Generating unusual answers to Rorschach Figures
Aziz-Zadeh et al. (2013)	1	13	Creative Cognition	Creative Synthesis Task
Ellamil et al. (2012)	1	15	Creative Cognition	Designing book cover illustrations
^∗^Huang et al. (2013)	1	28	Divergent Thinking	Imaging pictures visually cued
Kowatari et al. (2009)	2	20	Artistic creativity	Designing new pens

*Studies marked with an asterisk are based on the divergent thinking approach, but participants were instructed to generate only one response rather than providing many different responses to the same problem (standard divergent thinking task requires ideational fluency).*

*Studies on artistic creativity enrolled professional artists, such as pianists, or subjects with artistic training.*

### Activation Likelihood Estimation

The coordinates from studies identified in 24 published papers were used for ALE, which models the uncertainty in the localization of activation foci using Gaussian probability density distributions (Fox et al., 2014). In other words, ALE assesses the overlap between foci by modeling the probability distributions centered at the coordinates of each one (Eickhoff et al., 2009). This is calculated at each voxel and results in a thresholded ALE map. The probabilities of all activation foci in a given experiment were combined for each voxel, yielding a modeled activation map (Turkeltaub et al., 2012). ALE scores quantified the convergence across experiments at each particular location in the brain. ALE scores were compared against an empirical null distribution reflecting a random spatial association between the model activation maps (Eickhoff et al., 2009).

We performed a general ALE meta-analysis on the foci derived from the selected studies (**Table 1**). The coordinates of the foci were taken from the original papers. A total of 492 foci were reported in 45 experiments involving 1007 participants.

We also performed three separate ALE analyses to assess the neural correlates of creativity in different cognitive domains (i.e., Musical, Verbal, and Visuo-spatial). The experimenters (Maddalena Boccia, Laura Piccardi, Liana Palermo, Raffaella Nori, and Massimiliano Palmiero) independently classified the studies. Studies including different cognitive domains were excluded from these analyses: the data from these studies were included in the general analysis but not in the further analyses. Separate ALE analyses were performed on (1) 13 studies assessing musical creativity (219 participants, 197 activation foci), (2) 24 studies assessing verbal creativity (575 participants, 207 activation foci), and (3) six studies assessing visuo-spatial creativity (164 participants, 52 activation foci).

The ALE meta-analysis was performed using GingerALE^1^ 2.3.1 with MNI coordinates (Talairach coordinates were automatically converted into MNI coordinates by GingerALE), according to Eickhoff et al.’s (2009) procedure. The Full-Width Half-Maximum (FWHM) value was automatically computed, as this parameter is empirically determined (Eickhoff et al., 2009). The thresholded ALE map was corrected for multiple comparisons using False Discovery Rate (FDR), at a 0.05 level of significance. Moreover, a minimum cluster size of 200 mm^3^ was chosen. The ALE results were registered on an MNI-normalized template using MRICRO. Hereafter the link to access MRICRO^2^

### Tasks and Contrasts Taken into Account

Regarding the musical domain, participants were instructed to improvise music of various kinds (Classical, Jazz, etc.) on simple piano keyboards designed for usage in the scanner. In particular, music improvisation performed by modification of a melodic template was contrasted with the memorized improvisation previously made (Bengtsson et al., 2007); music improvisation using notes within the C major scale with over-learned tracks (Limb and Braun, 2008); lyric improvisation using an 8-bar instrumental track at 85 beats per minute with the memorized lyrics (Liu et al., 2012); melody improvisation and pseudo-random key presses production with sight reading of a musical score (de Manzano and Ullén, 2012); rhythmic (note choice constrained) and melody (note choice free) improvisation with or without metronome click synchronization with the reproduction of simple pre-learned 5-note patterns (Berkowitz and Ansari, 2008); rhythm improvisation (based on a rhythm listened to) with the reproduction of the rhythm heard (Villarreal et al., 2013); music improvisation (tonal, atonal, happy, and fearful) with the rest condition (Pinho et al., 2014).

Regarding the verbal domain, the ability to find alternative uses for an object, such as ‘a brick’ (AUT), was contrasted with fluency objects for location (indicating different objects in a specific place, such as an office), using the AU vs. 2-back memory (Abraham et al., 2012) as inclusive mask; the AUT with the fluency object for location and both these tasks with 1- and 2-back memory tasks in males vs. females and vice versa (Abraham et al., 2014). Furthermore, new ideas (unknown) provided by the AUT were contrasted with old ideas (recruited from the memory), and both new/old ideas vs. zero (Benedek et al., 2014b), whereas common or uncommon uses of objects were contrasted with a perceptual baseline task (Chrysikou and Thompson-Schill, 2011). The AUT was also contrasted with the object characteristic task (find typical characteristics of objects), the Incubation-AUT (reflect on ideas and elaborate them) with the standard AUT and vice versa, the Stimulation-AUT (the stimulus word was presented with three other people’s ideas) with the standard AUT (Fink et al., 2010); the Stimulation-AUT (original/common ideas of other people) with the control condition (the stimulus word was presented with two pseudowords) and the Stimulation-AUT (original) with Stimulation-AUT (common), (Fink et al., 2012). In addition, the generation of metaphor was contrasted with the production of literal responses (synonyms of adjectives; Benedek et al., 2014a); the unusual or creative generation of verbs in response to specific nouns with the generation of verbs that first came to mind (Seger et al., 2000) or with uncreative verbs (Green et al., 2015); the generation of creative stories from three words with uncreative stories and the generation of stories from unrelated words with stories from related words in the set (Howard-Jones et al., 2005); the creative writing was contrasted with the copying of a given text (Shah et al., 2013); the generation of inventive conceptions (biological functional feature associations) with ordinary conceptions (non-biological functional feature associations) and with baseline (Zhang et al., 2014).

Regarding the visuo-spatial domain, the generation of unique responses to Rorschach’s test was contrasted with the generation of frequent responses (Asari et al., 2008); the creative synthesis task (combination of three shapes, such as a circle, an ‘8’ and a ‘C, to form a creative object) with the reconstruction of a shape by combining three distinct stimuli in which the original shape was trisected (Aziz-Zadeh et al., 2013); the generation of ideas while designing book cover illustrations with the evaluation of ideas generated (Ellamil et al., 2012); the generation of creative pictures based on given visual clues with the generation of uncreative figures not necessarily unique (Huang et al., 2013). Finally, Kowatari et al. (2009) explored the neural correlates of designing of a new pen in experts and novices.

### Results

#### Neural Correlates of Creativity

**FIGURE 1:**
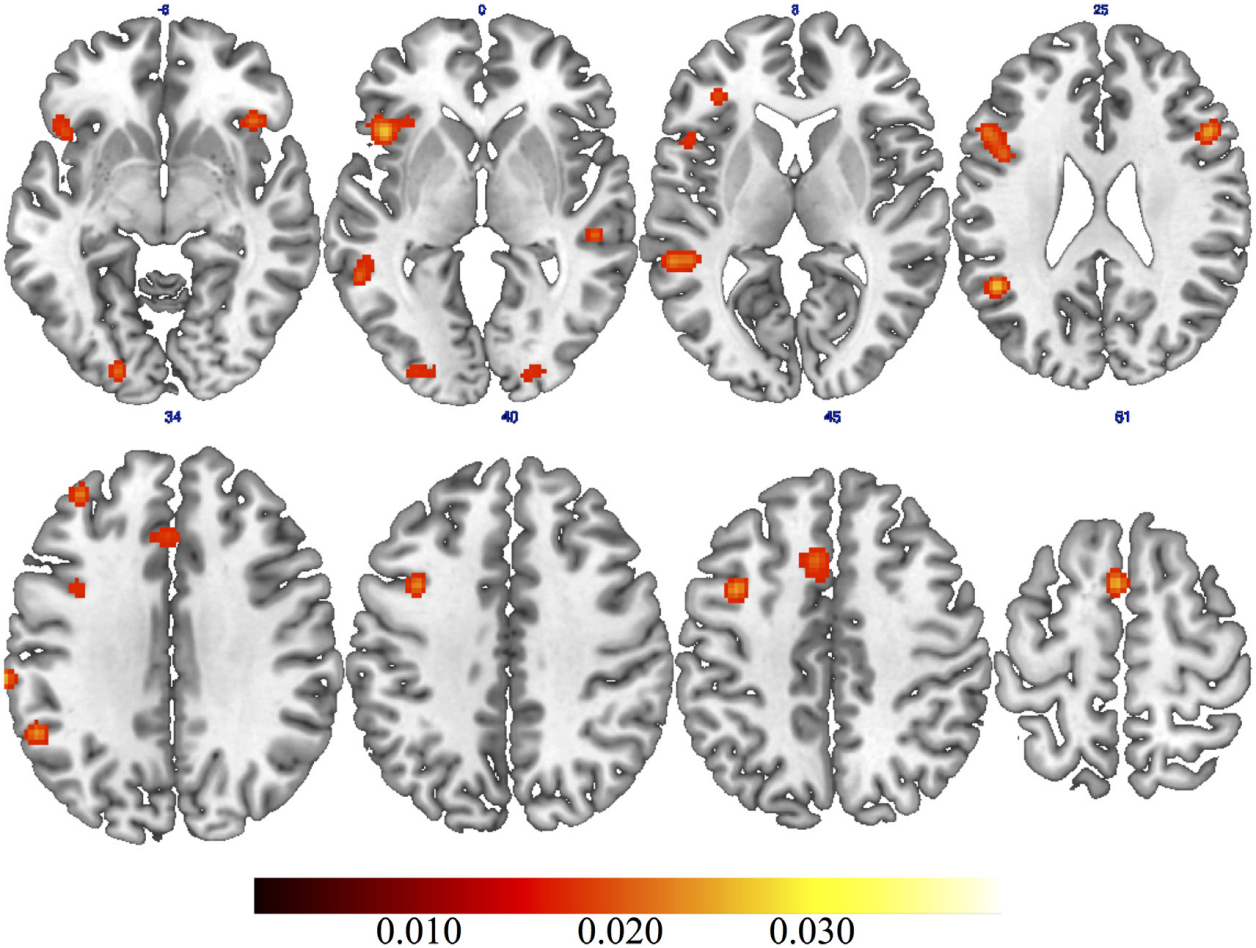
**Results of general activation likelihood estimation (ALE) meta-analysis on creativity**.

The general ALE meta-analysis showed clusters of activations ranging from the occipital to the frontal lobe (**Table 2**), in both the left and the right hemispheres (**Figure 1**). Specifically, we found consistent activations in the bilateral inferior, middle and medial frontal gyri as well as in the bilateral middle occipital gyrus. In the left hemisphere we found consistent activations in the precentral gyrus, superior frontal gyrus, inferior parietal lobule, supramarginal gyrus, insula, cingulate gyrus, and middle temporal gyrus. In the right hemisphere we found clusters of activation in the superior temporal gyrus. We also found consistent activation in the right posterior cerebellum.

**Table 2:** Regions showing consistent activations across fMRI studies of creativity, as resulting from the general activation likelihood estimation (ALE) analysis.

Region	Hem^a^	BA^b^	ALE extrema value	Cluster size^c^	*x*^d^	*y*	*z*
Insula	L	13	0.027	5896	-44	18	-2
Middle frontal gyrus	L	6	0.024		-38	6	44
Middle frontal gyrus	L	6	0.023		-40	2	50
Inferior frontal gyrus	L	9	0.022		-44	8	20
Middle frontal gyrus	L	9	0.022		-50	18	24
Precentral gyrus	L	6	0.019		-52	4	48
Precentral gyrus	L	6	0.018		-38	6	32
Precentral gyrus	L	44	0.017		-48	16	8
Insula	L	13	0.016		-34	24	0
Superior frontal gyrus	L	6	0.026	2408	-6	18	48
Medial frontal gyrus	L	6	0.025		-2	8	60
Middle temporal gyrus	L	22	0.025	2032	-48	-40	6
Supramarginal gyrus	L	40	0.026	1568	-48	-52	24
Supramarginal gyrus	L	40	0.024		-54	-50	32
Middle frontal gyrus	R	9	0.027	872	48	18	26
Middle occipital gyrus	L	18	0.022	840	-22	-90	-4
Middle occipital gyrus	L	18	0.019		-30	-90	2
Inferior orbitofrontal cortex/insula	R	47	0.022	776	42	24	-8
Inferior frontal gyrus	R	47	0.016		40	32	-12
Cingulate gyrus	L	32	0.021	760	-2	28	32
Medial frontal gyrus	R	6	0.040	520	12	-14	78
Middle occipital gyrus	R	18	0.019	424	22	-90	-2
Inferior parietal lobule	L	40	0.031	368	-66	-28	32
Posterior cerebellum	R		0.022	320	6	-50	-38
Middle frontal gyrus	L	8	0.024	272	-36	44	36
Middle frontal gyrus	R	6	0.018	272	28	0	50
Inferior frontal gyrus	L	46	0.019	248	-36	36	8
Superior temporal gyrus	R	22	0.021	224	52	-28	0

*^a^Hemisphere; ^b^Brodmann’s areas if applicable; ^c^Cluster volume (mm^3^); ^d^MNI coordinates.*

#### Neural Correlates of Musical Creativity

**FIGURE 2:**
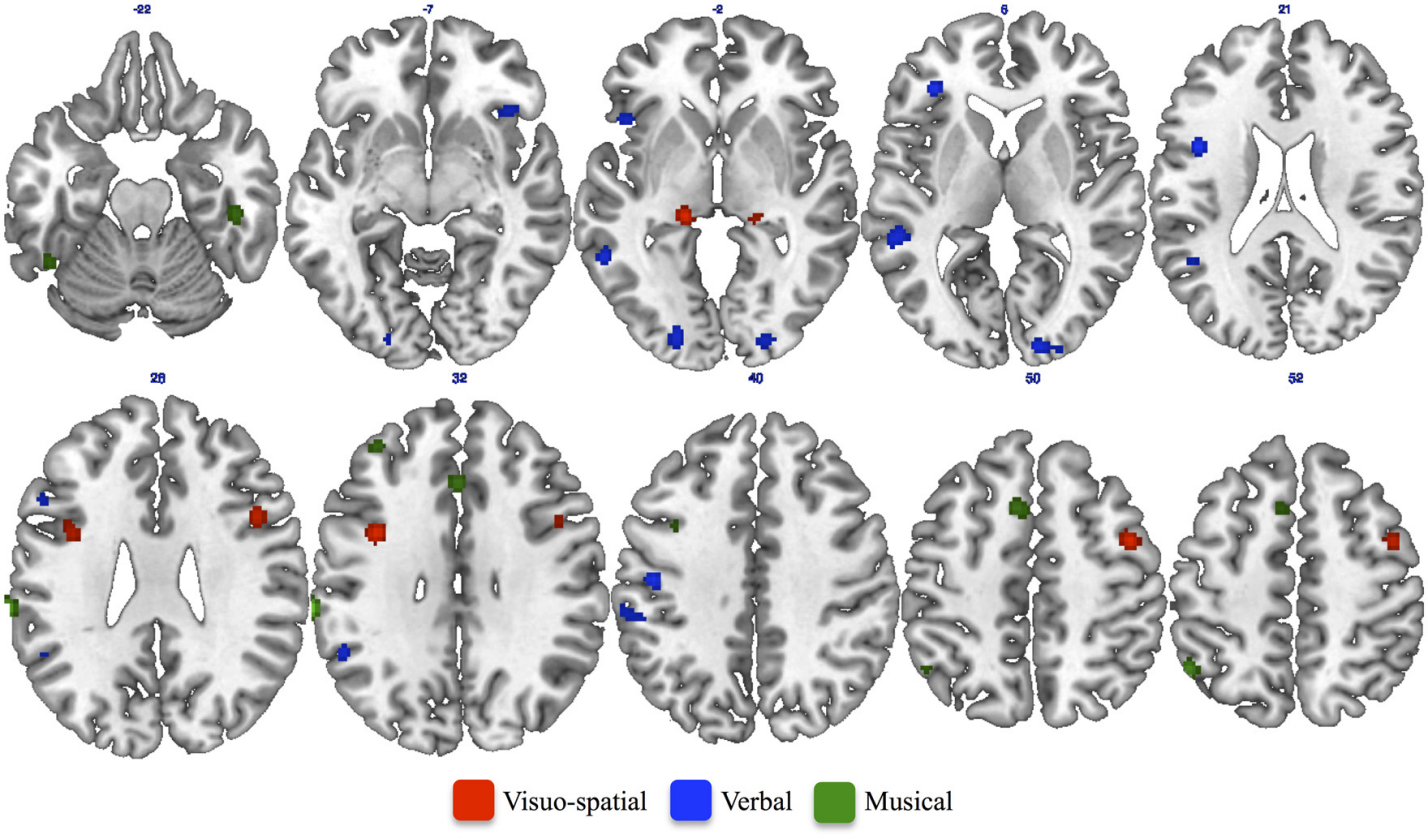
**Results of single ALE meta-analysis on studies assessing Musical (green patches), Verbal (blue patches), and Visuo-spatial (red patches) creativity**.

The ALE meta-analysis performed on studies assessing musical creativity showed clusters of activation in the bilateral medial frontal gyrus (**Figure 2**). Consistent activations were also found in the cingulate gyrus, middle frontal gyrus and inferior parietal lobule in the left hemisphere (**Figure 2**). In the right hemisphere we found activation in the postcentral and fusiform gyri (**Figure 2**). Furthermore, we found cerebellar activations, in the anterior lobe of the left hemisphere and in the posterior lobe of the right hemisphere (**Table 3**).

**Table 3:** Regions showing consistent activations across fMRI studies of musical creativity.

Region	Hem^a^	BA^b^	ALE extrema value	Cluster size^c^	*x*^d^	*y*	*z*
Medial frontal gyrus	R	6	0.040	640	12	-14	78
Posterior cerebellum	R		0.022	512	4	-50	-38
Medial frontal gyrus	L	32	0.018	456	-6	16	48
Inferior parietal lobule	L	40	0.030	368	-66	-30	30
Middle frontal gyrus	L	8	0.024	360	-36	44	36
Fusiform gyrus	R	20	0.016	344	46	-28	-22
Cingulate gyrus	L	32	0.017	336	0	28	32
Inferior parietal lobule	L	40	0.025	272	-48	-56	54
Middle frontal gyrus	L	6	0.015	240	-36	8	42
Anterior cerebellum	L		0.015	200	-46	-52	-22
Postcentral gyrus	R	7	0.020	200	20	-48	76

*^a^Hemisphere; ^b^Brodmann’s areas if applicable; ^c^Cluster volume (mm^3^); ^d^MNI coordinates.*

#### Neural Correlates of Verbal Creativity

The ALE meta-analysis performed on studies assessing verbal creativity showed clusters of activations mainly located in the left hemisphere (**Table 4**). We found consistent activation in the inferior and middle frontal gyri, middle and superior temporal gyri, inferior parietal lobule, postcentral and supramarginal gyri, middle occipital gyrus, and insula in the left hemisphere (**Figure 2**). We also found activation in the inferior frontal gyrus and lingual gyrus of the right hemisphere (**Figure 2**) as well as in the right posterior cerebellum.

**Table 4:** Regions showing consistent activations across fMRI studies of verbal creativity.

Region	Hem^a^	BA^b^	ALE extrema value	Cluster size^c^	*x*^d^	*y*	*z*
Middle temporal gyrus	L	22	0.014	1360	-54	-38	4
Superior temporal gyrus	L	22	0.014		-56	-40	10
Middle temporal gyrus	L	22	0.014		-56	-48	0
Lingual gyrus	R	17	0.014	928	18	-94	4
Middle temporal gyrus	L	39	0.014	560	-56	-56	10
Superior temporal gyrus	L	22	0.013	520	-46	-52	22
Supramarginal gyrus	L	40	0.013		-52	-50	32
Middle occipital gyrus	L	18	0.015	472	-20	-90	-4
Inferior frontal gyrus	L	45	0.013	440	-46	20	0
Middle frontal gyrus	L	9	0.014	416	-52	20	24
Insula	L	13	0.016	336	-42	6	20
Postcentral gyrus	L	3	0.016	336	-46	-16	42
Insula	R	47	0.012	280	40	24	-8
Inferior frontal gyrus	R	47	0.011		36	22	-10
Inferior frontal gyrus	L	46	0.015	272	-34	36	6
Inferior parietal lobule	L	40	0.012	264	-60	-30	38
Inferior parietal lobule	L	40	0.011		-52	-34	40
Posterior cerebellum	R		0.014	248	32	-82	-32
Inferior frontal gyrus	L	47	0.011	200	-26	30	-20
Inferior frontal gyrus	L	47	0.010		-36	28	-20

*^a^Hemisphere; ^b^Brodmann’s areas if applicable; ^c^Cluster volume (mm^3^); ^d^MNI coordinates.*

#### Neural Correlates of Visuo-Spatial Creativity

The ALE meta-analysis performed on studies assessing visuo-spatial creativity showed clusters of activation in the middle and inferior frontal gyri of the right hemisphere as well as in the bilateral thalamus (**Table 5**). We also found consistent activation in the left precentral gyrus (**Figure 2**).

**Table 5:** Regions showing consistent activations across fMRI studies of visuo-spatial creativity.

Region	Hem^a^	BA^b^	ALE Extrema value	Cluster size^c^	*x*^d^	*y*	*z*
Precentral gyrus	L	6	0.013	584	-38	4	32
Thalamus	L		0.013	464	-16	-28	-2
Middle frontal gyrus	R	6	0.013	464	44	2	50
Inferior frontal gyrus	R	9	0.011	368	45	12	28
Thalamus	R		0.009	232	18	-30	-2
Thalamus	R		0.009		24	-28	2

*^a^Hemisphere; ^b^Brodmann’s areas if applicable; ^c^Cluster volume (mm^3^); ^d^MNI coordinates.*

## Discussion

The main aim of the present study was to find converging evidence for a multi-componential neural system for creativity based on open-ended mental problems in different cognitive domains. First of all, we performed a general ALE analysis to give a general picture of the brain networks involved in creativity. Then three separate ALE analyses were performed in order to assess the neural correlates of creativity in Musical, Verbal, and Visuo-spatial domains. We found a wide network of areas, ranging from the occipital to the frontal lobe, in both left and right hemispheres. A functional specialization was found within this network for different types of creativity, confirming Dietrich and Kanso’s (2010, p. 822) idea that distinguishing different types of creativity is valuable “to make creativity tractable in the brain.” This is also in line with the hypothesis of the existence of a functional multi-componential system in the human brain for creative thinking. Even if previous quantitative meta-analyses on creativity have been made (Gonen-Yaacovi et al., 2013), to our knowledge this is the first meta-analysis clearly disentangling the brain regions underpinning musical, verbal, and visuo-spatial creativity, based on the generation of creative solutions to open-ended problems.

Specifically, the recruitment of executive functions is crucial for creativity. The activations found in the left anterior cingulate cortex (ACC), as well as in the bilateral inferior frontal gyri and middle frontal gyri (DLPFC), may be strictly connected to “more executive” aspects of creativity, since these areas are activated during conditions of high cognitive control (Miller and Cohen, 2001). In particular, activation of the DLPFC is correlated with effortful problem-solving, monitoring, and focused attention (Ashby et al., 1999). DLPFC also plays a key role in the selection process (Nathaniel-James and Frith, 2002), being linked to extra working memory load due to keeping in mind different alternatives (Bookheimer, 2002) and comparing many different stimuli. Thus, although these processes were not directed tested, it is not surprising that the DLPFC was found to be consistently activated during Musical (right hemisphere), Verbal (left hemisphere), and Visuo-spatial (right hemisphere) creativity, which generally require effortful problem solving, focused attention, selection process and working memory.

Concerning specific-domain activations, we found that verbal creativity consistently activated the left inferior frontal gyrus. Since verbal creativity has been reported to require the ability to integrate distant semantic concepts or ideas in a new fashion (Benedek et al., 2012; Benedek and Neubauer, 2013; Zhang et al., 2014), by means of semantic retrieval and selection of stored knowledge (Thompson-Schill et al., 1997; Seger et al., 2000; Badre et al., 2005; Moss et al., 2005; Badre and Wagner, 2007), these processes may well have entailed the activation of the left inferior frontal gyrus. On the other hand, attentional processes (Zhang and Li, 2012) and successful response inhibition (e.g., Aron et al., 2014) may entail activations of the right inferior frontal gyrus. However, these activations were found both during verbal and visuo-spatial creativity, but not during musical improvisation, which seems to rely more upon response inhibition. Thus, although one might claim that the inhibition of competitive responses during the creative act is supported by the right inferior frontal gyrus, the functional role of this area while performing on musical, verbal or visuo-spatial creativity tasks needs to be more fully addressed.

The high cognitive control during musical and verbal creativity also induced activations of the left inferior parietal lobule. Hemispheric specialization has been proposed for this area. Specifically, verbal attention (Jordan et al., 2001), and language-related processes with a focus on semantic and phonological issues (Vigneau et al., 2006) were found to recruit the left inferior parietal lobule, which also belongs to the default mode network (Buckner et al., 2008). Furthermore, although the activations of the left inferior parietal lobule, supramarginal gyrus and insula shown by the general ALE analysis might also indicate multimodal sensory processing and the representation of subjective experience during spontaneous creativity (Csikszentmihalyi, 1996), further study is necessary to better clarify this issue.

Interestingly, musical and visuo-spatial creativity activate regions involved in motor planning, such as the right supplementary and the left premotor cortices, probably indicating that a motor and temporal planning is crucial for creative musical improvisation (Brown et al., 2006; Bengtsson et al., 2007; Berkowitz and Ansari, 2008; Limb and Braun, 2008; Pinho et al., 2014), as well as in the visuo-spatial rotation of objects (Milivojevic et al., 2009) during visuo-spatial creativity.

The posterior activations found in the temporal (left middle temporal gyrus and right superior temporal gyrus) and occipital (bilateral middle occipital gyrus) lobes across different creativity domains deserve consideration. According to Dietrich (2004), the posterior cortices are essential for creativity, being the sites of long-term memory storage (e.g., Gilbert, 2001) and being connected to the prefrontal cortex. Therefore, given that creativity relies on an associative mode of processing, heightening focused attention to stored knowledge that facilitates efficient retrieval and recombination of existing information (Fink et al., 2012), the activation of the posterior cortices may be the neural correlates of such processes. Moreover, given that these areas have a pivotal role in generating mental images (Kosslyn and Thompson, 2003), these results could also support the relationship between creative processes and mental imagery. Specifically, according to the Perceptual Anticipation Theory, mental images arise when an individual “anticipates perceiving an object or scene so strongly that a depictive representation of the stimulus is generated in early visual cortex” (Kosslyn and Thompson, 2003; p. 724). Thus, it may be that information stored in the long-term memory is selectively retrieved and used to form mental images, which subtend the generation of creative ideas. Other brain areas are then needed to explore and finalize ideas in different cognitive domains. In this direction, musical creativity showed the activation of the right fusiform gyrus and parietal postcentral gyrus, whereas verbal creativity showed the recruitment of the left middle and superior temporal gyri, right lingual gyrus, left middle occipital gyrus, and left parietal postcentral gyrus.

Finally, the right posterior cerebellum was recruited in both verbal and musical creativity, indicating searching processes for appropriate responses (Seger et al., 2000). Such a result suggests that the cerebellum may have an important role in creativity. Indeed, by permitting previously executed movements, which have been proved to be advantageous, the cerebellum allows individual motor sequences to be consolidated into more complex patterns underlying the generation of novel creative outcomes (Cotterill, 2001). However, due to the lack of systematic studies on this issue, the specific role of the cerebellum in creativity is still unclear.

## Conclusion

The results of the present meta-analysis of fMRI studies of creatvity based on open-ended problems in musical, verbal, and visuo-spatial domains suggest that different domains of creativity roughly correspond to a higher activation in functionally specialized brain areas. In general, frontal areas seem to be crucial for idea generation, although there are slight differences across creativity domains. Activation of the DLPFC was found in all creativity domains under investigation, whereas the inferior frontal gyrus was recruited consistently in verbal creativity and weakly in visuo-spatial creativity. This finding suggests that creativity relies on the activation of the prefrontal cortex, which likely works as an executive engine, managing attentional recourses, retrieving, and selecting appropriate information. Future studies should take into account the ‘gateway hypothesis’ (Burgess et al., 2007), which highlights the role of the rostral prefrontal cortex on attending behavior that enhances the ability to notice change in the environment (stimulus-oriented cognition) as well as on self-generated or maintained representations (stimulus-independent cognition). Focusing on this latter ability, the lateral rostral prefrontal cortex would work as a ‘gateway’ between the process of selection of actions or thought operations and the stimulus-independent attending system, ensuring that activation of representations is less affected by sensory input. This is exactly the case of creativity, which is mainly based on stimulus-independent processes, retrieval of information from the memory and selection of the most appropriate responses to satisfy specific criteria, such as originality and appropriateness. Unfortunately the gateway hypothesis has never been directly tested by means of a paradigm investigating creativity.

Interestingly, part of the default network (the left inferior parietal lobule) and different temporal, parietal, and occipital areas were found to be recruited while performing on musical and verbal creativity, but not when performing on visual creativity. Also the right posterior cerebellum was activated during both musical and verbal creative processes. Thus, the present meta-analysis would seem to indicate that musical and verbal creativity share common areas that involve attentional, searching, and associative modes of processing of stored knowledge from the posterior cortices, and temporarily represent information in the working memory buffer with the aid of prefrontal areas. On the contrary, visuo-spatial creativity would appear to rely consistently on the perception and manipulation of visual stimuli, such as the rotation of shapes; in this direction, visuo-spatial creativity strongly yielded activations in the bilateral thalamus and premotor cortices, the former being involved in relaying sensory information, the latter in finalizing in a top–down fashion the goal-directed planning of novel ideas. However, it is surprising that visuo-spatial creativity did not produce the activation of any temporal, parietal, and above all occipital regions, considering that the recruitment of these areas was reported in various studies of visual creativity (e.g., Huang et al., 2013). Probably, given the scarcity of the number of experiments (6–164 participants, 52 activation foci) belonging to the visuo-spatial domain, the ALE analysis did not highlight these results, thus making the findings somewhat less reliable.

Therefore, generally speaking, creativity seems to emerge when the prefrontal cortex, posterior temporal, and parietal areas are recruited. This is also confirmed by studies with dementia patients (for a review, see Palmiero et al., 2012), who show a decline in divergent thinking and artistic creativity when these areas are damaged. On the other hand, it is possible that, since all the studies we included in the ALE meta-analysis checked for early visuo-spatial features by using well-designed control conditions, the ALE statistics only showed brain areas more related to general visuo-spatial creative processes, such as premotor regions supporting mental rotation of stimuli, rather than to visual properties *per se*. This is also true in the case of musical creativity, in which we found no activation of the auditory cortex. It should be stressed, though, that all the included studies compared activations during a creative condition (usually assessed by means of musical improvisation) with those during a control condition (usually assessed by means of the reproduction of conventional pieces). The failure to find any activation of the auditory cortex is likely due to the fact that this area is generally involved in musical and auditory processes but it is not directly entailed in musical creativity.

Regarding the lateralization issue, the unbalanced number of studies across the domains could account for the activations mainly of the left hemisphere in the general ALE analysis. However, looking at the separate ALE analyses, musical and verbal creativity showed predominant activations in the left hemisphere, whereas visuo-spatial creativity in the right hemisphere, but a clear laterality effect was not found. This suggests that inter-hemispheric interaction is required in all domains of creative processes (Dietrich and Kanso, 2010) and supports the idea that creative processes are subtended by different brain areas and functional specialized brain regions rather than by a specific brain area.

Finally, on the basis of the findings outlined above, creativity appears to be a multifaceted process, involving different mental functions, and studied using different approaches and tasks. Although the number of experiments and critical contrasts in each category separately is, at present, insufficient for statistical testing, in the future, in order to reach more reliable conclusions, neural correlates of creativity should be studied considering the interaction among the domains, approaches and tasks used. A higher number of studies will also allow for a contrast analysis and a conjunction analysis among different creativity domains, now impossible due to the paucity of the studies. Different creativity domains should also be explored, such as dance and scientific innovation. Of course this meta-analysis was not aimed at determining the specific executive, default and memory processes supported by cerebral regions during creativity. Further studies should therefore explore whether and how idea generation and evaluation emerge in different creativity domains.

## Conflict of Interest Statement

The authors declare that the research was conducted in the absence of any commercial or financial relationships that could be construed as a potential conflict of interest.

## Footnotes

1 http://www.brainmap.org

2 http://www.mccauslandcenter.sc.edu/mricro/index.html

∗ Citations marked with an asterisk were included in the meta-analysis.
